# The use of progress testing

**DOI:** 10.1007/s40037-012-0007-2

**Published:** 2012-03-10

**Authors:** Lambert W. T. Schuwirth, Cees P. M. van der Vleuten

**Affiliations:** 1 Flinders Innovation in Clinical Education, Flinders University, Adelaide, Australia; 2 Department of Educational Development and Research, Maastricht University, Maastricht, the Netherlands; 3 Chair Department of Educational Development and Research, Maastricht University, Maastricht, the Netherlands

**Keywords:** Assessment, Educational, Activities, Educational, Learning, Collaboration

## Abstract

Progress testing is gaining ground rapidly after having been used almost exclusively in Maastricht and Kansas City. This increased popularity is understandable considering the intuitive appeal longitudinal testing has as a way to predict future competence and performance. Yet there are also important practicalities. Progress testing is longitudinal assessment in that it is based on subsequent equivalent, yet different, tests. The results of these are combined to determine the growth of functional medical knowledge for each student, enabling more reliable and valid decision making about promotion to a next study phase. The longitudinal integrated assessment approach has a demonstrable positive effect on student learning behaviour by discouraging binge learning. Furthermore, it leads to more reliable decisions as well as good predictive validity for future competence or retention of knowledge. Also, because of its integration and independence of local curricula, it can be used in a multi-centre collaborative production and administration framework, reducing costs, increasing efficiency and allowing for constant benchmarking. Practicalities include the relative unfamiliarity of faculty with the concept, the fact that remediation for students with a series of poor results is time consuming, the need to embed the instrument carefully into the existing assessment programme and the importance of equating subsequent tests to minimize test-to-test variability in difficulty. Where it has been implemented—collaboratively—progress testing has led to satisfaction, provided the practicalities are heeded well.

## Introduction

Progress testing is becoming increasingly popular both in the Netherlands and internationally [1–9] after having been used for a long time only in those institutions where it was invented: the University of Missouri-Kansas City School of Medicine and Maastricht University in the Netherlands [10, 11]. The rapid spread of the concept, however, is not surprising because a longitudinal approach to assessment has an intrinsic appeal. It is intuitively more logical to assess students repeatedly and combine their results on these assessments to make predictions about future competence and/or performance. It is similar to a child’s development monitoring programme. In such programmes the child is weighed and measured at regular intervals and the outcomes are compared with population mean growth curves in order to detect and remedy problems as early as possible. This is probably also the reason why such an abundance of developmental and research papers on this topic have found their way to the literature in recent decades.

But it is not as straightforward as it looks; introducing progress testing involves not only a change in thinking about assessment but also an academic cultural change. Even more so, when collaboration on progress testing is sought; in such situations openness, non-competitiveness, exchange and mutual trust are essential. The purpose of this paper is to summarize the most important expectations and to accompany them with experiences from actual practice.

## What is progress testing?

The many different descriptions of progress testing largely converge on the principle of longitudinal, repeated assessment of students’ functional knowledge. Often, a number of tests are set per academic year, each consisting of a large number of questions pitched at graduate level functional (relevant) knowledge. Each of these tests is sat by students of multiple or all year classes, and the results of each individual test are combined in a compensatory way to form the basis for a promotion decision at the end of the year. The test is comprehensive in that it consists of questions covering a broad domain of relevant medical knowledge, and it is organizationally founded on centralized test production, review, administration and analysis. Our description here is intentionally general because there are various different implementations possible, and more detailed descriptions are provided in the literature [1, 3, 5, 7, 11, 12].

## Expectations and practicalities of progress testing

### Reduction of examination stress

Because progress tests are longitudinal measurements it is assumed that students will experience less examination stress, because a one-off bad result cannot undo a series of good results [11–13]. The—formative—collaborative progress test in the German speaking countries is even largely student led [5] and largely based on a bottom-up development. When McMaster formally evaluated their newly introduced progress test, a fair proportion (39%) of the students reported very little to no stress, a larger proportion (48%) reported limited stress and only a small proportion (27%) indicated moderate to high stress [3]. Yet, there is another side of the coin; if a single bad result cannot ruin a good series it is likewise difficult to make up for a bad series. This is particularly an issue when students are about to graduate, and all other examination requirements have been met, but they still have poor progress test results. A bad series of progress test results then has to be remediated, and one can safely assume that each of the subsequent sittings is a stressful event for those students, and in our experience in practice they are.

### Repeat examinations become unnecessary

Another reported advantage of progress testing is that it renders resit examinations unnecessary. Resits are a burden for the organization; they have to be good quality examinations for only a small number of students. Also, they can lead students to adopt a minimalistic study approach; why study hard when there are always the resits [14]? But again, the side effect is that students in trouble have no quick repeat possibility, and may need to defer their graduation for some time, with very negative financial consequences.

### Positive influence of student learning

Undisputed is the positive influence on student learning. This is actually why progress testing was originally developed [10, 11], and in the various implementations there is evidence to underpin this positive effect. In McMaster the test led students to study more continuously and to build a better knowledge base, preparing them better for the national licensing examinations [15]. The positive effect of progress testing can be seen clearly from curves showing the growth of medical knowledge. Not only can it be seen that the amount of functional knowledge grows continuously (without huge peaks and troughs), but also that the basic knowledge is retained over the year classes [3, 5, 11, 12, 16–18]. Though such continuous growth occurred even if non-problem based learning or non-integrated curricula used progress testing [8, 9], growth curves were more irregular (with more peaks and troughs) when progress testing was not a summative element of the programme [19].

However, no assessment method can exert its influence on student learning in a vacuum; it always works in the context of the rest of the assessment programme [14, 20]. When progress testing was introduced in Maastricht and block tests were made formative, students changed their focus to continuous self-directed learning, but when the—mastery orientated—block test was made summative again, many students reverted to short-term memorization despite the progress test remaining unchanged.

### Better predictive validity

Another assumed advantage is that longitudinal data collection is more predictive of future competence/performance than one-off measurements. For this, choices have to be made with respect to how to combine the information of subsequent tests. Some schools opt for a more continuous approach [3] and use regression techniques to make predictions, others acknowledge the discrete nature of the information and combine qualifications [5, 11, 13]. We feel that both are defensible choices but that equating or controlling for difficulty variation is a more pressing issue. Langer et al. [21] have elaborated on this problem and have suggested some solutions. Unfortunately, most solutions are not practical in a medical school setting [21–25]. Equating techniques may be impossible to apply in the normal routine (the use of anchor items may induce students to memorize old tests) and item response theory (IRT) may simply require too much pretesting to be practical either. More feasible statistical smoothing techniques such as Bayesian models [24] or moving average techniques [22, 23] on the other hand may be too difficult to explain, especially to students whose original score has to be downgraded by the statistical procedures. This would seriously limit the already rocky base for university acceptance of the concept of progress testing.

### Better reliability of decisions

Finally, longitudinal combination of results adds to the reliability of the decision. Research in the 1980s and onwards [26, 27] has made it clear that the sampling properties are much more important for reliability than how well structured the test is [28]. It is logical to assume that the combined result of four tests of 200 items each (in the case of Maastricht) is better than one big test, and a large test distributed over various occasions has better sampling than a one-off large test. Ricketts et al. [29] quantified this using generalizability theory and reported the standard errors of measurement (SEM) as a trade-off between number of items per test and number of tests per year. Their findings indicate that two tests of 200 items per year produce more reliable results (lower SEMs) than four tests of 100 items each, or even five tests of 100 items. So although there is value in having more occasions it is not simply more-occasions-is-better.

Another important discussion point in reliability is that most progress tests employ a correct-minus-incorrect (formula) scoring system. This is necessary because the tests are also administered to junior students. It is not considered desirable that our junior students—not being able to answer most of the questions—would be forced to guess on many items. Therefore, a question-mark option has to be offered with formula scoring. Whether or not this decreases the reliability of progress test scores is open to debate. When the test is taken under formula scoring conditions the number of correct reliabilities is higher—the difference being roughly 0.20 (unpublished results of the interuniversity progress test in the Netherlands)—but experimental studies where scores under formula scoring and number-right conditions were compared showed better reliabilities for the formula scoring [30, 31].

### Comprehensive tests are less predictable for the test-savvy students

The comprehensiveness of the test content is often seen as an advantage too, because specific strategic revision does not work (what would you study if the whole of medical knowledge is sampled from?) [3, 11, 15, 32, 33]. So the longitudinality influences the imminence and threatening nature of the test [34] and the comprehensiveness influences the nature of assessable material in such a way that the best preparation is continuous learning [34]. But there is, again, another side to this, as it has to be very clear what the nature of assessable material is. In other words, what is relevant functional knowledge and what is not? This is an issue that still remains unresolved. It will take a feasible operationalization of ‘relevance’ for test writers, reviewers and users to be able to agree on the relevance of each item.

### Curriculum independence and collaboration

A final advantage is the progress test’s curriculum independence. The fact that it is designed to test knowledge at graduate level makes it perfect for joint production, joint administration and joint research. The many emerging collaborations [1, 2, 5–9, 35] are proof of this. This is not to say that collaboration is easy or comes naturally. Schools for example are used to having complete ownership of their assessment material and collaboration means that they have to give up some of that ownership. Also coordination of test administrations, mutual dependency and division of labour may present considerable infrastructural and administrative hurdles [6].

## Epilogue

Progress testing is definitely an important addition to the available assessment methods. It has become clear that in a programme of assessment it should not be used to replace current methods but to add to them [20, 36, 37]. Good knowledge of the pros and cons, the indications and contraindications, is a prerequisite for good usage of progress testing, and we hope this paper has contributed to this.

## Essentials

- Progress testing is a longitudinal test approach based on equivalent tests given at fixed intervals with the intention to assess the development on functional knowledge or competence
- The biggest advantage of progress testing is that it minimizes test-driven learning strategies
- Combining the results on the repeated tests increases both the reliability of pass–fail decisions and its predictive validity
- A major concern with progress testing is ensuring the equivalence of the individual tests
- When progress testing is used in a collaborative fashion—sharing test production and administration—it is not only more cost-effective but also a rich source for continuous benchmarking and quality improvement
